# A detailed characterization of the hyperpolarization-activated “funny” current (*I*_f_) in human-induced pluripotent stem cell (iPSC)–derived cardiomyocytes with pacemaker activity

**DOI:** 10.1007/s00424-021-02571-w

**Published:** 2021-05-02

**Authors:** Federica Giannetti, Patrizia Benzoni, Giulia Campostrini, Raffaella Milanesi, Annalisa Bucchi, Mirko Baruscotti, Patrizia Dell’Era, Alessandra Rossini, Andrea Barbuti

**Affiliations:** 1 The Cell Physiology MiLab, Department of Biosciences, Università degli Studi di Milano, Via Celoria 26, 20133 Milano, Italy; 2 Present Address: Department of Anatomy and Embryology, Leiden University Medical Center, Einthovenweg 20, 2333ZC Leiden, The Netherlands; 3 Present Address: Dipartimento di Medicina Veterinaria, Università degli Studi di Milano, Via dell’Università 6, 26900 Lodi, Italy; 4 Cellular Fate Reprogramming Unit, Department of Molecular and Translational Medicine, University of Brescia, viale Europa 11, 25123 Brescia, Italy; 5 Institute for Biomedicine, Eurac Research, Affiliated Institute of the University of Lübeck, Viale Druso 1, 39100 Bolzano, Italy

**Keywords:** Funny current, Human-induced pluripotent stem cells (hiPSC), Sinus node, HCN channels, Pacemaker

## Abstract

Properties of the funny current (*I*_f_) have been studied in several animal and cellular models, but so far little is known concerning its properties in human pacemaker cells. This work provides a detailed characterization of *I*_f_ in human-induced pluripotent stem cell (iPSC)–derived pacemaker cardiomyocytes (pCMs), at different time points. Patch-clamp analysis showed that *I*_f_ density did not change during differentiation; however, after day 30, it activates at more negative potential and with slower time constants. These changes are accompanied by a slowing in beating rate. *I*_f_ displayed the voltage-dependent block by caesium and reversed (*E*_rev_) at − 22 mV, compatibly with the 3:1 K^+^/Na^+^ permeability ratio. Lowering [Na^+^]_o_ (30 mM) shifted the *E*_rev_ to − 39 mV without affecting conductance. Increasing [K^+^]_o_ (30 mM) shifted the *E*_rev_ to − 15 mV with a fourfold increase in conductance. pCMs express mainly *HCN4* and *HCN1* together with the accessory subunits CAV3, KCR1, MiRP1, and SAP97 that contribute to the context-dependence of *I*_f_. Autonomic agonists modulated the diastolic depolarization, and thus rate, of pCMs. The adrenergic agonist isoproterenol induced rate acceleration and a positive shift of *I*_f_ voltage-dependence (EC_50_ 73.4 nM). The muscarinic agonists had opposite effects (Carbachol EC_50_, 11,6 nM). Carbachol effect was however small but it could be increased by pre-stimulation with isoproterenol, indicating low cAMP levels in pCMs. In conclusion, we demonstrated that pCMs display an *I*_f_ with the physiological properties expected by pacemaker cells and may thus represent a suitable model for studying human *I*_f_-related sinus arrhythmias.

## Introduction

Rhythmicity of cardiac contractions derives from the spontaneous electrical oscillations of the sinoatrial node (SAN). In these cells, at the end of the repolarizing phase of the action potential, a slow diastolic depolarization (DD) drives the membrane potential to the threshold for firing the next action potential. Although the DD is due to a complex interplay of various ionic mechanisms, the pacemaker “funny” current (*I*_f_) plays a pivotal role [18]. The *I*_f_, described for the first time in 1979 in rabbit sinoatrial cardiomyocytes, owes its name to its unusual property of being activated upon hyperpolarization. f-channels are non-selective channels conducting a mixed Na^+^ and K^+^ current that display a dual voltage and ligand gating, being activated upon membrane hyperpolarization and direct binding of cAMP. In mammals, f-channels are the product of the HCN gene family consisting of 4 isoforms (HCN1-4). In the SAN of many species, HCN4 is the most abundant isoform followed by HCN1 and to a lesser extent HCN2 [8, 11, 13]. The importance of f-channels to cardiac rhythmicity is demonstrated by the fact that mutations in *HCN4* have been found in patients with several sinus arrhythmias, such as sinus bradycardia, inappropriate sinus tachycardia, sinus node disease but also with atrial fibrillation and ventricular non-compaction [17]. Moreover, alterations in either the physiological levels of HCN channel or in *I*_f_ current properties have been linked to other arrhythmias and cardiomyopathy [9, 35].

The majority of studies addressing the physiological and pathological role of *I*_f_ have been performed in rabbit and murine SAN cells [12, 16]. This choice derives from the difficulty of retrieving human SAN tissue. Indeed, while atrial and ventricular cardiomyocytes can be isolated from small biopsies during various types of surgeries, the SAN, due to its function and dimension is practically inaccessible. So far, only few studies analysed HCN channel expression in the human SAN [13, 28] and only one study described the properties of *I*_f_ in three cells isolated from a diseased human SAN [39].

The possibility to differentiate pluripotent stem cells into cardiomyocytes has opened a new opportunity to obtain SAN-like cells. Previous studies have demonstrated that SAN-like cells derived from mouse embryonic stem cells (mESC) show an *I*_f_ current with properties very similar to those of the native mouse SAN cells [5, 36]. Similarly, human induced pluripotent stem cells (hiPSC) have made easily available a source of human pacemaker cardiomyocytes, giving us the unique opportunity to analyse the properties of the human *I*_f_ current. Here we present a full functional characterization of the *I*_f_ current recorded from regularly and spontaneously beating cardiomyocytes, here dubbed pCMs (pacemaker cardiomyocytes), at different time points of differentiation.

## Material and methods

### Maintenance of hiPSCs lines and cardiac differentiation

All the hiPSC lines used were from healthy donors. We used previously characterized and published hiPSC lines [1, 9] derived both from females and male donors of different ages. Moreover, a new line has been generated from blood cells of a healthy male donor (age 52) following an informed consent, in agreement with the declaration of Helsinki and its use was approved by the ethical committee of the Università degli Studi di Milano (nr. 29/15). Human iPSC lines were maintained on Matrigel-coated plates in TeSR-E8 medium (Stem Cell Technologies). Cells were passaged using Tryple Express (Thermo Fisher Scientific) every 4 days and seeded at the density of 20,000 cells/cm^2^. Cardiac differentiation was induced at least 30 passages after the generation of the lines and were used up to passage 140. Within this interval, we did not observe any significant variation in either the differentiation capacity or in cardiomyocytes yield. Cardiac differentiation was carried out on hiPSC monolayers using the PSC Cardiomyocyte Differentiation Kit (Thermo Fisher Scientific), following the manufacturer’s instructions. Briefly, when iPSCs reached 70–80% of confluency, cardiomyocyte differentiation medium A was added; after 48 h, medium was replaced with cardiomyocyte differentiation medium B, and after other 48 h, medium was replaced with the cardiomyocyte maintenance medium (CMM) that was refreshed every 2 days. hiPSC-derived cardiomyocytes were maintained in culture for 15, 30, or 60 days.

### Quantitative reverse transcriptase PCR (qRT-PCR) analysis

Total RNA was isolated using TRIzol (Thermo Fisher Scientific). GoScript™ Reverse Transcription System (Promega) was used to synthesize cDNA following the manufacturer’s instructions. For each gene, qRT-PCR was performed on technical duplicates or triplicates from at least 4 independent experiments, using 10 ng of cDNA with the iQTMSYBR® Green Supermix (Bio-Rad) using the iCycler Bioer System (BIOER). Expression data were analysed using 2^(-ΔCT) method using β-actin (*ACTB*) as housekeeping gene. Gene expression levels were normalized to cardiac Troponin-T (*TNNT2*) levels to account for differences in cardiomyocytes yield among various differentiation experiments. Primers used are given below.

**Table:** 

*ACTB*	F: CACTCTTCCAGCCTTCCTTC	R: AGTGATCTCCTTCTGCATCCT
*TNNT2*	F: AAGCCCAGGTCGTTCATGCCC	R: CTCCATGCGCTTCCGGTGGA
*HCN1*	F: TGAAGCTGACAGATGGCTCTT	R: CTGGCAGTACGACGTCCTTT
*HCN2*	F: CTGATCCGCTACATCCATCA	R: AGATTGCAGATCCTCATCACC
*HCN3*	F: TGGATCCTACTTTGGGGAGA	R: ATGGTCCACGCTGAGTGAGT
*HCN4*	F: AACAGGAGAGGGTCAAGTCG	R: ATCAGGTTTCCCACCATCAG
*CAV3*	F: CGAGGACATAGTCAAGGTGGAT	R: AGAAGGAGATGCAGGCGAAC
*KCNE2 *(MiRP1)	F: ACTGCATAGCAGGAGGGAAGC	R: TCAGCATCAACTTTGGCTTGG
*ALG10* (KCR1)	F: CTGGCTTGTACCTGGTGTCA	R: GGATACTTGAGGCAGCCTTGT
*DLG1* (SAP97)	F: GGTCACGCCTCTCTTCAGAC	R: CACACACCTTGCCCTAGCC

### Immunofluorescence (IF) staining and Western blot analysis

hiPSC-CMs were fixed in 4% paraformaldehyde and incubated in a blocking PBS solution with 0.3% Triton X-100 (Sigma-Aldrich) and 3% Donkey serum, for 45 min. Antibodies used are reported below. Nuclei were stained with 0.5 µg/ml DAPI. Western blot analyses were carried out loading 120 µg of protein extracts; proteins were separated by SDS-PAGE and transferred onto PVDF membranes. Chemiluminescence signals were acquired with the Chemidoc system (BioRAD) after membrane incubation with SuperSignal™ West Pico/Fempto PLUS Chemiluminescent Substrate (Thermo Fisher Scientific). Membranes were incubated with primary antibodies overnight at 4 °C and secondary antibodies for 1 h at RT under agitation. Mouse anti-cardiac Troponin (Abcam, clone 1C11, 1:1000), rat anti-HCN4 (Abcam, 1:2000), mouse anti-HCN1 (Termofisher, 1:1000), rabbit anti-CAV3 (Abcam, 1:500), appropriate secondary antibodies conjugated to HRP (Jackson ImmunoResearch, 1:10,000) for WB, and Alexa −488 and −594 coniugated (Jackson ImmunoResearch, 1:600) for IF were applied. Densitometric analyses of WB bands for HCNs isoforms were performed using Image J software.

### Electrophysiological analysis

hiPSC-derived cardiomyocytes were isolated at day 15, 30, or 60 with tripsyn-EDTA (Sigma) and plated on fibronectin (Corning)-coated dish. Electrophysiological experiments were performed using either the ruptured or perforated patch-clamp configuration at 36 ± 1 °C on pCM. The extracellular Tyrode solution (pH 7.4) contained (mM): 137 NaCl, 5 KCl, 2 CaCl_2_, 1 MgCl_2_, 10 D-glucose, 10 Hepes–NaOH. Patch pipettes had a resistance of 4–7 MΩ and 10-12 MΩ, for voltage- and current-clamp recordings, respectively, when filled with intracellular-like solution (pH 7.1) containing (mM) the following: 120 KCl, 20 Na-HEPES, 10 MgATP, 0.1 EGTA-KOH, 2 MgCl_2_.

The *I*_f_ current was recorded from isolated pCM adding BaCl_2_ (1 mM) and MnCl_2_ (2 mM) to the Tyrode solution (CTRL condition) to minimize interference from K^+^ and Ca^2+^ currents. *I*_f_ was activated from a holding potential (hp) of −30 mV by applying 10-mV hyperpolarizing voltage steps from −35 to −125-mV long enough to reach steady-state activation, followed by a fully activating step at −125 mV. Steady-state current density was calculated as the ratio between current intensity and cell capacitance at all voltages. Activation curves were obtained from normalized tail currents and fitted to the Boltzmann equation:

$$y=1/\left(1+\mathrm{exp}\left(\left(V-{V}_{1/2}\right)/s\right)\right)$$

where *V*_1/2_ is the half-activation voltage and *s* the inverse slope factor. Activation time constants (*τ*) were calculated by fitting traces to a single exponential curve in the range −75/–125 mV, after an initial delay.

Fully activated current density–voltage (*I*/*V*) relations from day 30 pCMs were determined as previously published [20]. Briefly, *I*_f_ was recorded from a hp of −35 mV by applying pairs of steps at −125 mV (all channels open) and +20 mV (all channels closed) each one followed by test steps in the range −120/ +20 mV (in 20 mV increments); fully activated current was determined as the arithmetical difference between initial current amplitude elicited by test steps at the same voltage. To block the *I*_*f*_ current, 2 mM CsCl was added to the control solution. The potassium-dependence was studied increasing the external K^+^ concentration to 30 mM using the following external solution (in mM): 110 NaCl, 1.8 CaCl_2_, 0.5 MgCl_2_, 30 KCl, 1 BaCl, 2 MnCl_2_, 5 HEPES NaOH (pH 7.4). Sodium-dependence was studied decreasing the external Na^+^ concentration to 30 mM, using the following external solution (mM): 30 NaCl, 107 NMDG-Cl, 5 KCl, 2 CaCl_2_, 1 MgCl_2_, 10 D-glucose, 10 Hepes–NaOH (pH 7.4).

To dissect the effect of autonomic agonists on *I*_f_ and rate, isoproterenol (from 10 to 3000 nM) or carbachol (from 1 to 1000 nM) has been added to either Tyrode or control solution from concentrated stock solutions. The voltage shifts were calculated as previously reported [4] by applying hyperpolarizing pulses from − 30 mV (hp) to a voltage close to *V*_1/2_ and compensating the differences in current amplitude caused by drugs perfusion by manually changing the amplifier holding command. Shifts were plotted against drug concentrations and fitted to the Hill equation:

$$y=Y{\max}\raisebox{1ex}{\it x}^{\raisebox{1ex}{\it n}} \!\left/ \! \raisebox{-1ex}{\it k}^{\raisebox{-1ex}{\it n}}{\raisebox{-1ex}{+{\it x}}}^{\raisebox{-1ex}{\it n}}\right.$$

where *Y*max represents the maximal shift, *k* the EC_50_, and *n* the Hill coefficient.

## Statistics

Data were analysed with Clampfit 10 (Molecular Devices) and Origin Pro 9 (OriginLab). Normal distribution of data points was assessed using the Kolmogorov–Smirnov test; groups were compared with one-way ANOVA followed by pairwise comparison using Fisher’s test. *P* < 0.05 defines statistical significance. Normally-distributed data are presented as Mean ± Standard Error of the Mean (SEM).

## Results

### Kinetic properties of ***I***_f_

**Fig. 1:**
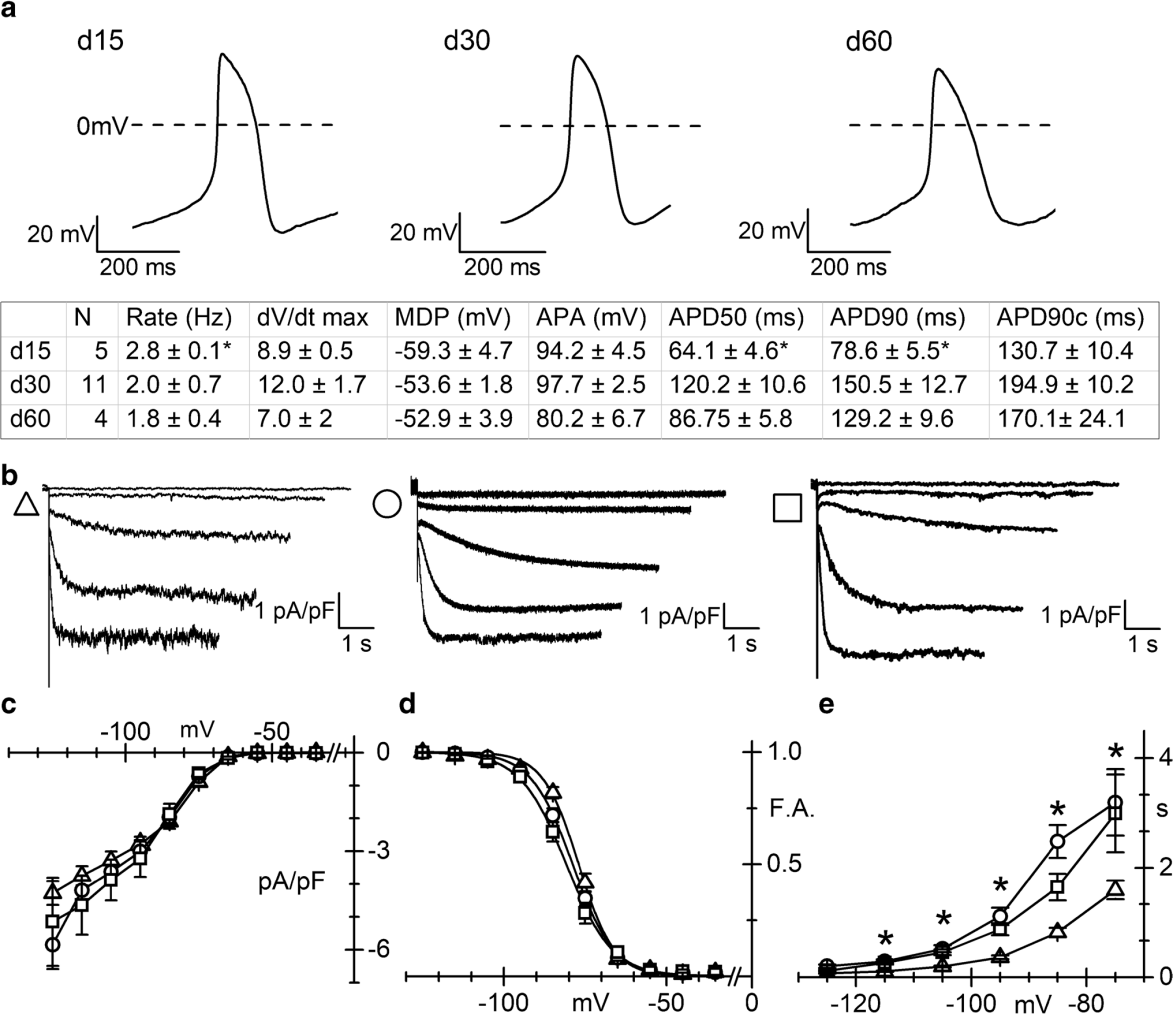
Action potential properties and *I*_f_ density and kinetic in pCM. **a** (Top) Representative spontaneous action potentials recorded from d15, 30, and 60 pCMs, as indicated; (Bottom) Summary table of the AP parameters: APA, action potential amplitude; MDP, maximum disatolic potential; APD, action potential duration at 50% (APD50) or 90% (APD90) of repolarization; APD90c, rate corrected APD90. **b** Representative traces of the *I*_f_ current density recorded in the range −35/ −115 mV (20 mV increment), at the three different differentiation stages (d15, triangles; d30, circles; d60, squares throughout the figure)**. c** Plot of mean *I*_f_ current density–voltage relations obtained at the different time points. Mean values at −115 mV were as follows: − 3.75 ± 0.29 pA/pF (*n* = 19), − 4.19 ± 0.45 pA/pF (*n* = 21), and − 4.64 ± 0.90 pA/pF (*n* = 13). **d** Plot of the mean *I*_f_ activation curves; *V*_1/2_ and inverse-slope factors were as follows: − 76.5 ± 0.71* mV and 4.8 ± 0.36 (*n* = 19), − 79.0 ± 0.8 mV and 5.6 ± 0.36 (*n* = 21), − 81.2 ± 1.37 mV and 6.4 ± 0.47 (*n* = 13) at days 15, 30, and 60, respectively. Asterisk indicates *P* = 0.041 day 15 vs 30 and *P* = 0.0011 day 15 vs 60. **e** Plots of *I*_f_ activation time constant (*τ*) in the range − 75 to − 125 mV. Mean *τ* values at − 75 mV were as follows: 1.6 ± 0.2 s* *n* = 19; 3.2 ± 0.61 s *n* = 21; 2.9 ± 0.71 s *n* = 13, at days 15, 30, and 60, respectively**.** Asterisk indicates *P* = 0.0423 day 15 vs 30 and *P* = 0.0454 day 15 vs and 60

Differentiation of hiPSC into cardiomyocytes is usually monitored by the appearance of spontaneously beating activity in the culture dishes, which strongly indicates the presence of a proportion of spontaneously contracting pacemaker cells. Following single cell isolation, we run patch clamp experiments only on cardiomyocytes showing regular pacemaker activity, here dubbed pCM (pacemaker cardiomyocytes). Figure 1a shows three representative action potentials (APs) recorded from single pCM at d15, 30, and 60, as indicated. AP parameters (Fig. 1 table) are in line with those previously reported for specifically selected nodal like cells [34]. Application of hyperpolarizing steps in the range −35/ −125 mV to pCM elicited time- and voltage-dependent inward currents with electrophysiological properties compatible with *I*_f_. Figure 1b shows representative traces recorded from pCMs at day 15 (triangle), day 30 (circle), and day 60 (square) of differentiation. Mean cell capacitance at the three time points was similar (20.3 ± 1.0 pF *n* = 19, 23.2 ± 1.9 pF *n* = 21, and 19.2 ± 2.1 pF *n* = 13 at days 15, 30, and 60, respectively). As shown by the steady-state *I*-*V* curves, current density did not vary significantly with time (Fig. 1c).

Analysis of the activation curves shows that at days 30 and 60, the voltage-dependence of *I*_f_ shifted slightly but significantly to more negative potentials than at day 15 (Fig. 1d). Finally, the analysis of activation time constant (*τ*) revealed that at day 15, the *I*_f_ current activated with significantly faster *τ* than at later differentiation days, in the range −75 to −115 mV (Fig. 1e).

### hiPSC-CMs express HCN isoforms and accessory subunits

In order to evaluate the subunit composition of the *I*_f_ current, we first investigated the expression of the HCN isoforms of beating cultures at d15, 30, and 60.

**Fig. 2:**
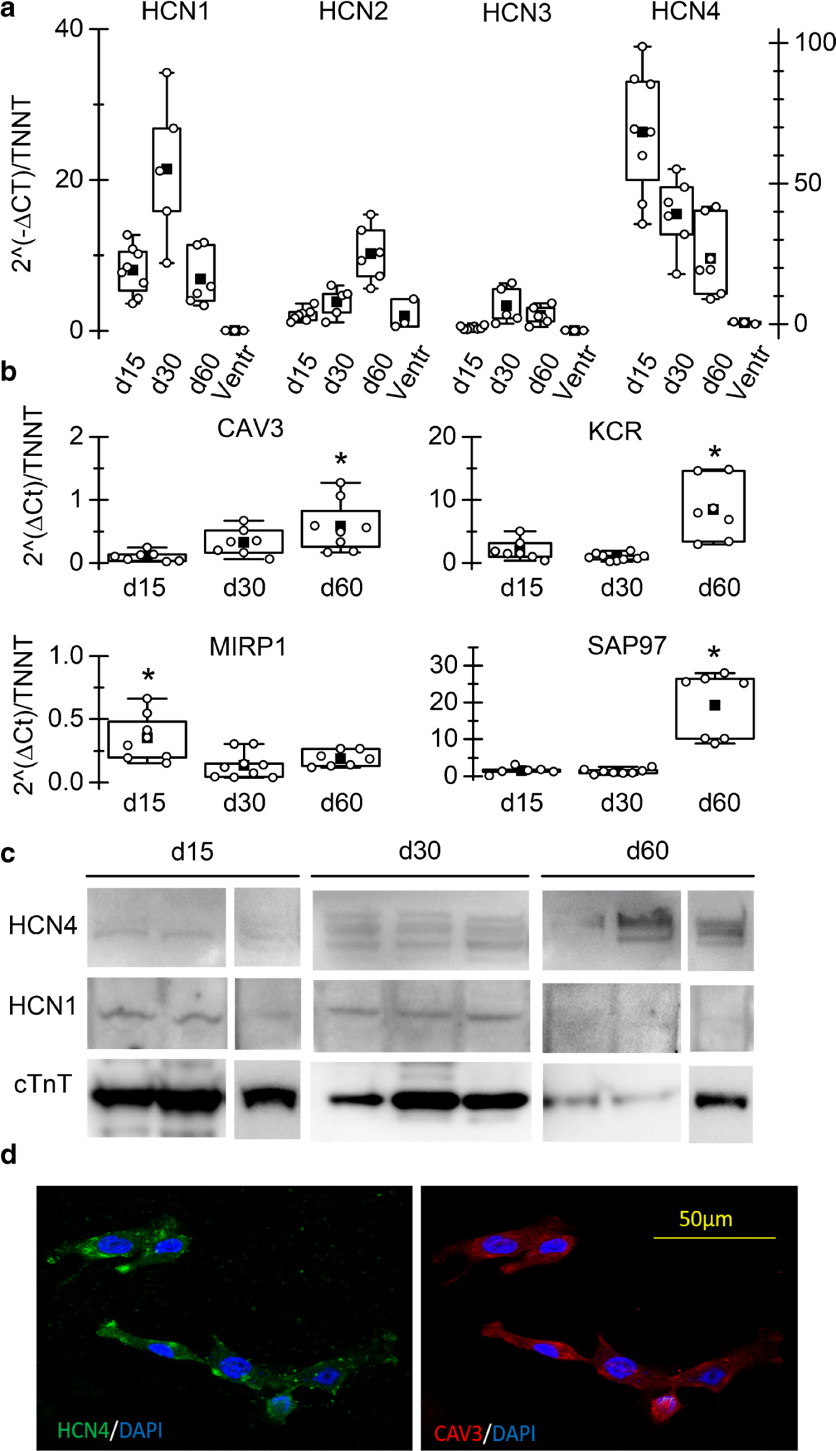
hiPSC-CMs express HCN isoforms and auxiliary proteins.** a** Box-plot showing qRT-PCR analysis of HCN1-4 genes in iPSC-CMs at d15, 30, and 60, as indicated; Troponin T expression was used as reference gene in each sample to normalize for cardiomyocyte yield in different differentiations. **b** qRT-PCR analysis of genes known to be f-channels auxiliary subunits (caveolin-3-CAV3; Alpha-1,2-Glucosyltransferase-KCR1; Discs large homolog 1-SAP97; and Potassium channel βsubunit-MIRP1) in beating cultures at the various time points. **c** WB analysis of HCN4 and HCN1 in three hiPS-CM cultures at the various time points. cTnT expression was used for estimating content in cardiomyocytes. **d C**onfocal microscopy image of hiPSC-CM showing co-expression of HCN4 (green) and caveolin-3 (red); nuclei were counterstained with DAPI (blue)

From box plots in Fig. 2a, it is clear that HCN4 and HCN1 are the most abundant isoforms expressed at all time points, in accordance with literature data on SAN cells of various species and on stem cell-derived SAN-like cells. HCN1 mRNA was more expressed at day 30 than at both day 15 and day 60; HCN2 expression increased significantly at day 60; HCN3 expression was almost absent at day 15 but increased at later time points remaining however low. Transcript levels of HCN channels in human ventricular samples are shown for comparison. In agreement with literature data, HCN4 and HCN1 are not expressed in the human ventricle.

We also analysed the expression of several known auxiliary proteins of f-channels (Fig. 2b) such as caveolin-3 (CAV3), KCR-1, MiRP-1, and SAP97 that, interacting with HCN, finely modulate their functional properties [7, 32, 35]. All genes were expressed in hiPSC-CMs.

Panel c shows Western blot analysis at each differentiation time-point for HCN1, HCN4, and cTnT. HCN4 is the prevalent isoform expressed at the protein level, at all time-points. It is worth noting that the densitometry analysis revealed that the HCN1/HCN4 ratio is significantly higher at day 15 than at the other time points (d15, 2.07*; day 30, 0,30; d60, 0,07. *n* = 3, *P* < 0.05 by Anova), pointing to a higher contribution of the fast-activating HCN1 isoform at d15 than at later time points. These data agree with and support the kinetics data shown in Fig. 1d and e.

Figure 2d shows a representative immunofluorescence image of hiPSC-CMs co-stained with anti-HCN4 and -caveolin-3 antibodies. As previously demonstrated, the co-expression of these two proteins is characteristic of pacemaker/SAN cardiomyocytes of different species [4, 33, 36].

These differences are compatible with a certain degree of functional maturation likely due to a variation in the context-dependence and/or stoichiometry of HCN subunits between d15 and d30. For this reason, the following analysis were carried out only at day 30, a good compromise between time of differentiation and maturity.

### Ionic nature of ***I***_f_

**Fig. 3:**
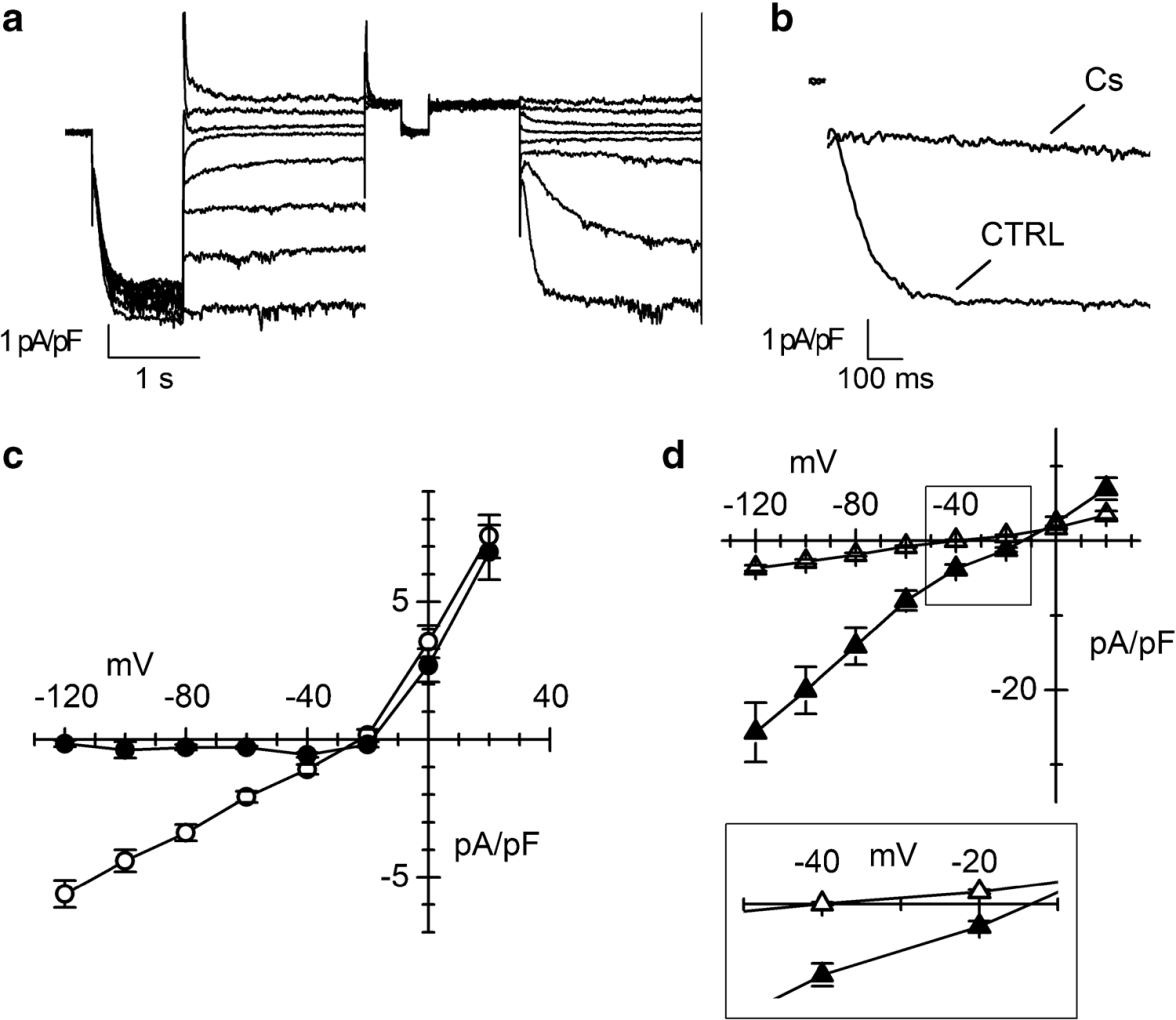
Characterization of the ionic nature of *I*_f_ current. **a** Representative normalized *I*_f_ traces recorded at day 30 of differentiation elicited by the protocol used to obtain the fully activated *I*-*V*. **b** *I*_f_ traces recorded at −125 mV before (CTRL) and during the superfusion of 2 mM Caesium (Cs) at day 30 of differentiation. **c** Mean fully activated *I*-*V* relations obtained without (empty circles, *n* = 27) or with 2 mM Cs (black circles, *n* = 20). **d** Mean fully activated *I*-*V* relations in the presence of 30 mM external potassium (filled triangles, *n* = 13) or 30 mM external sodium (empty triangles, *n* = 14). The inset shows a blow up of the *x*-axis to appreciate changes in the *E*_rev_

In Fig. 3a, representative current traces recorded applying the protocol described in the “Material and methods” section for obtaining the fully activated *I*-*V* relationship of *I*_f_ are shown. The reversal potential (*E*_rev_) estimated from the *I*-*V* was around − 22 mV (Fig. 3c), a value compatible with the mixed sodium and potassium permeability typical of *I*_f_. Addition of caesium (2 mM), a well-known blocker of the *I*_f_ current, to the extracellular solution almost completely suppressed the inward component of *I*_f_, especially at the most negative potentials (Fig. 3b and c), while did not affect the outward current, in agreement with the previously-reported voltage-dependent block [15].

In panel 3d, the effects of varying the external concentrations of Na^+^ and K^+^ on the fully activated *I*-*V* relations are shown. Changing the external potassium concentration from 5 to 30 mM increased conductance density more than fourfold (from 58.6 ± 4.9 to 256.0 ± 40.6 pS/pF, *n* = 30 and *n* = 14 respectively) and induced a positive shift of the *E*_rev_ (to about − 15 mV; Fig. 3d, filled triangles). When the *I*_f_ was recorded in the low sodium (30 mM) external solution, conductance did not change significantly (46.9 ± 4.8 pS/pF, *n* = 14) while the *E*_rev_ shifted to more negative voltages (around − 38 mV, Fig. 3d inset empty triangles). These effects are compatibles with previously reported data of *I*_f_ in rabbit SAN [20].

### Modulation of ***I***_f_ and spontaneous action potential rate by sympathetic and parasympathetic agonists

A well-studied modulatory mechanism of heart rate is based on a direct cAMP-dependent modulation of the funny current by autonomic neurotransmitters [19]. Here we assessed how *I*_f_ responded to different concentrations of both the β-adrenergic agonist isoproterenol (Iso) and the muscarinic agonist carbachol (CCh).

**Fig. 4:**
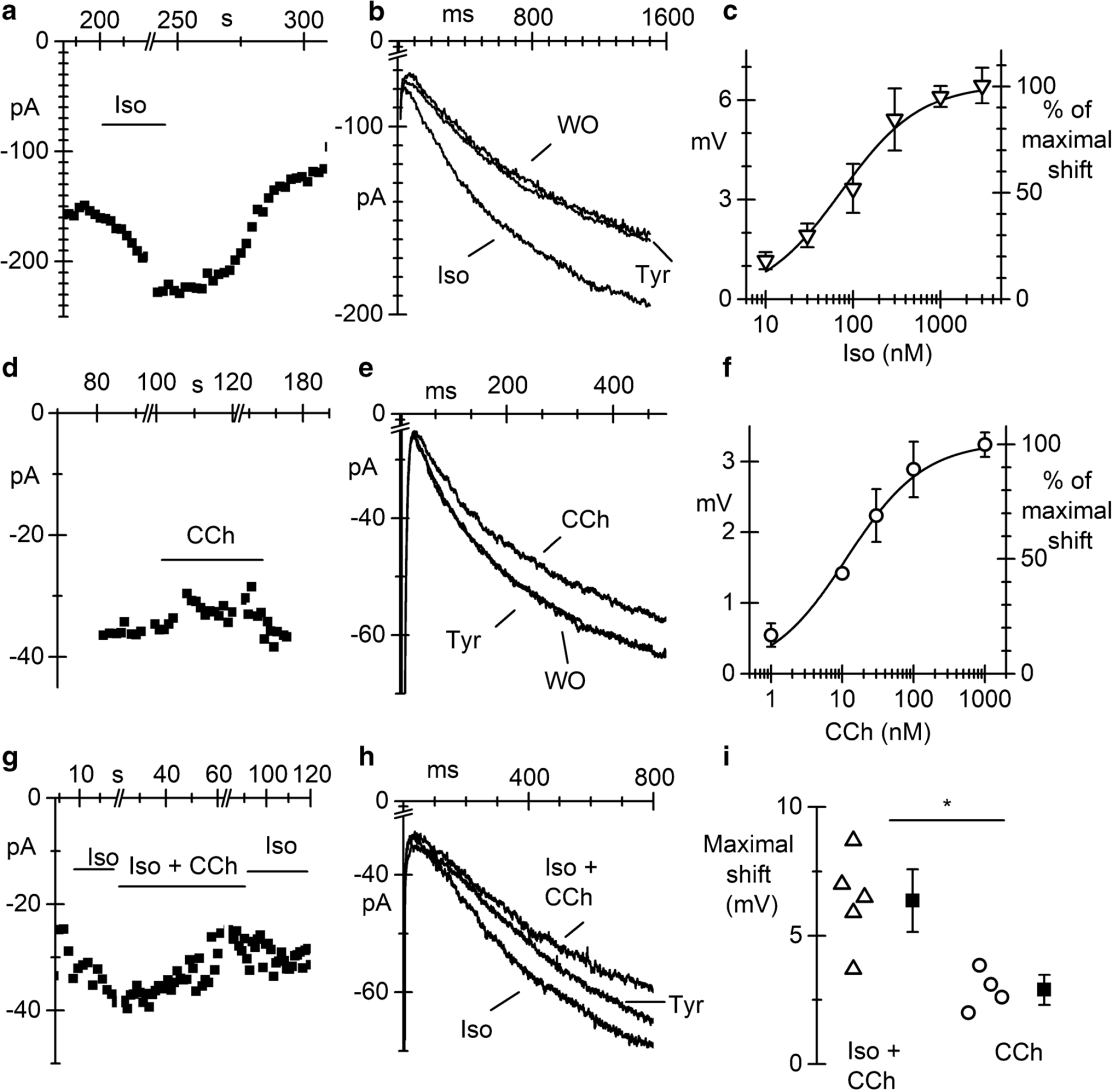
*I*_f_ current in pacemaker pCMs is modulated by sympathetic and parasympathetic agonists. **a** Time course of *I*_f_ current elicited by voltage steps at − 80 mV at d30 during perfusion of 1 μM isoproterenol (Iso). **b** Overlapped *I*_f_ traces in Tyrode (Tyr), during iso perfusion and after wash-out (WO). **c** Dose–response curve for isoproterenol. **d** Time course of *I*_f_ current elicited by voltage steps at −95 mV at d30 during perfusion of 100 nM carbachol (Cch). **e** Representative *I*_f_ current traces recorded before (Tyr), during (CCh), and after carbachol wash-out (WO). **f** Plot of dose–response curve for CCh. Continuous lines in panels **c** and **f** represent the best fitting to the Hill equation. **g** Time course of *I*_f_ current elicited by voltage steps at − 95 mV at d30 during perfusion of 100 nM iso alone and Iso + Cch 100 nM. **h** Overlapped *I*_f_ traces in Tyrode (Tyr), during superfusion of Iso (Iso), and during Iso+CCh.. **i** Dot plot of the shifts of the *I*_f_ activation curve caused by 100 nM CCh with or without pre-stimulation with 100 nM Iso, as indicated. *P = 0.0096

In Fig. 4a and b, the time course (a) of the *I*_f_ amplitude, elicited by voltage steps in the range −80/ −95 mV, before (Tyr), during (Iso) superfusion of 1 µM Iso, and after washout (WO) is shown together with representative traces, overlapped (b). As expected, isoproterenol reversibly increased *I*_f_ amplitude by shifting the activation curve to positive potentials. The shifts of the activation curve were calculated as described in the “Material and methods” section. The plot in Fig. 4c shows the dose–response curve of the shift, obtained with concentrations of Iso ranging between 10 and 3000 nM. Data points fitting with the Hill equation (see “Material and methods” section) gave a half-maximal effective concentration (EC_50_) of 73.4 nM and a Hill number of 0.96.

In Fig. 4d, e, and f, the time course, current traces, and dose–response curve of the shift obtained perfusing the parasympathetic agonist CCh at different concentrations (1 to 1000 nM) are shown. Perfusion of 100 nM CCh induced a reduction of the current amplitude (CCh in Fig. 4d and e) corresponding to a leftward shift of the activation curve of about 3 mV. Fitting of the dose–response curve with the Hill equation resulted in an EC_50_ of 11.6 nM and a Hill number of 0.8.

Since responses to CCh were smaller than expected from literature data [21, 41], we re-evaluated the effect of 100 nM CCh after previous stimulation of *I*_f_ with 100 nM Iso (Iso and Iso + CCh in Fig. 4e). Data in Fig. 4g–i show that under this experimental conditions, 100 nM CCh caused a mean shift of 6.4 ± 0.81 mV (*n* = 5), significantly higher than the 2.9 ± 0.34 mV shift caused by 100 nM CCh alone (*n* = 4). These data indicate that pCMs have low basal level of cAMP.

**Fig. 5:**
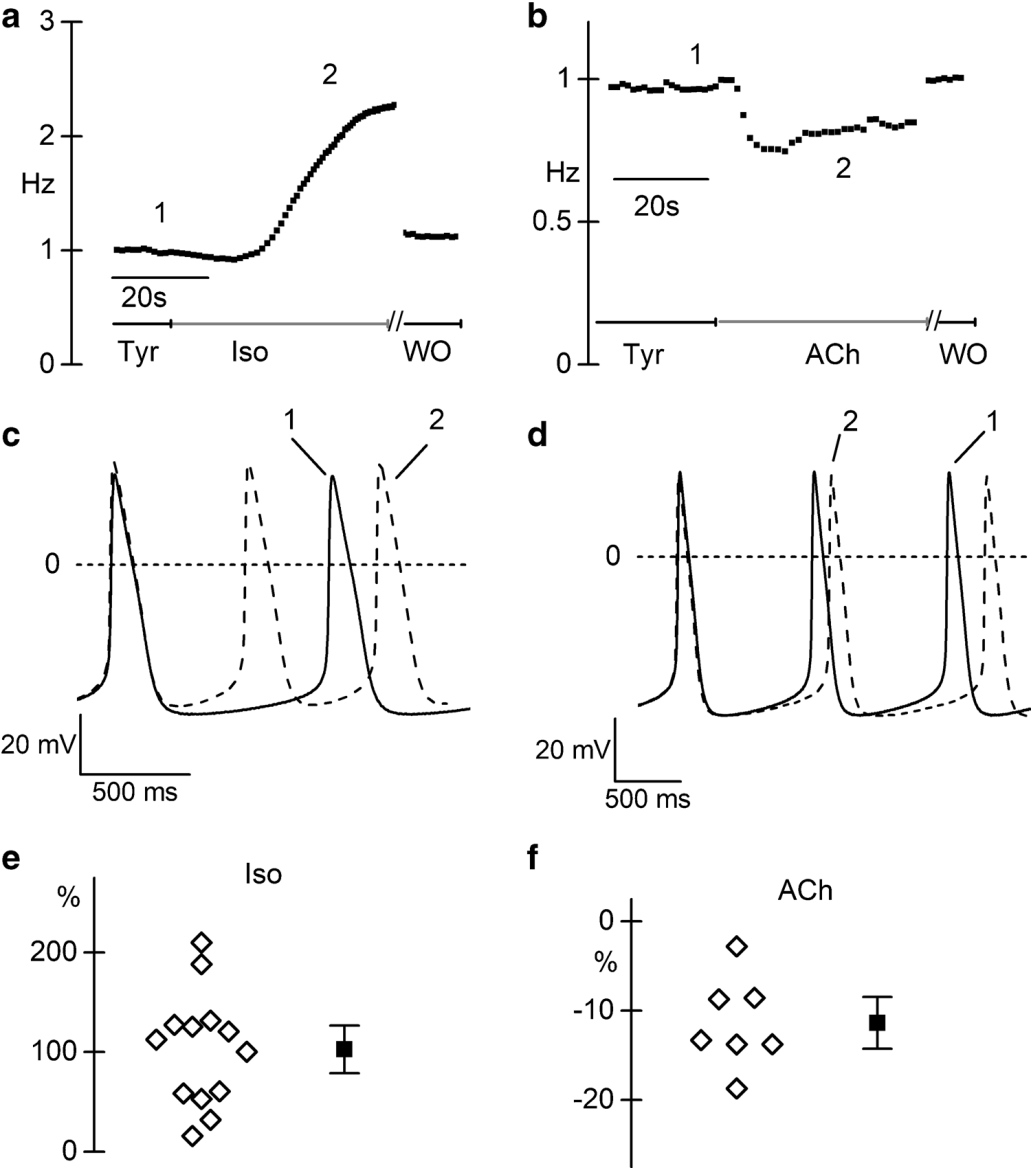
Spontaneous rate of pCMs is modulated by sympathetic and parasympathetic agonists. **a** Representative time course of the action potential rate in clusters of pCMs at 30 days of differentiation before, during 1 µM isoproterenol perfusion, and after washout, as indicated. **b** Representative time course of the action potential rate before, during, and after washout of 100 nM acetylcholine (ACh) perfusion. **c**, **d** Representative action potential traces recorded before (solid line) and during (dashed line) isoproterenol (**c**) and Ach (**d**) stimulation; traces correspond to points 1 and 2 in of the respective time courses. **e**, **f** Dot plot of the percentage change in firing rate during 1 µM Iso (**e**) and 100 nM Ach (**f**). Mean ± SEM value is reported as filled square and whiskers

We finally evaluated the effects of Iso (1 µM) and Ach (100 nM) on action potentials recorded from small aggregates of 30 day-old spontaneously beating pCMs. In Fig. 5a and b, representative time-courses of the beating rates before, during, and after Iso or Ach superfusion are plotted.

As expected, Iso accelerated, while ACh slowed the spontaneous beating rate. In panel 5c and 5d, stretches of action potential recordings in Tyrode (continuous line) and Iso or ACh (dashed line) are shown overlapped to highlight the changes in the slope of the diastolic depolarization (DD). Panels 5e and 5f show the dot plot of the % change in beating activity elicited by Iso (mean increase + 102.7 ± 16.0%, *n* = 13) and ACh (mean decrease − 12.5 ± 1.7%, *n* = 7). Coherently with the effect of the drugs on the *I*_f_, the slope of the DD significantly increased from 0.014 ± 0.002 to 0.029 ± 0.004 V/s during Iso superfusion (*P* = 0.0023; *n* = 13), but only slightly decreased with the muscarinic agonist (0.011 ± 0.002 V/s; *P* = 0.4731; *n* = 7). Again, the small effect of ACh on the DD is compatible with low intracellular cAMP levels.

## Discussion

In the last years, hiPSC-derived cardiomyocytes have been extensively used to model heart pathophysiology, in particular genetic arrhythmias such as long QT syndrome, CPVT, Brugada syndrome, and atrial fibrillation [9, 27, 31, 38]. Being patient- and pathology-specific, hiPSC-derived cardiomyocytes are the perfect tools for studying in vitro both the pathological mechanisms and drug response [24]. Moreover, this model overcome the problem of the paucity of the human cardiac cell availability.

The main limitation of iPSC-derived cardiac cell as a model to study working cardiomyocytes consists in their immature electrical phenotype. A functional marker of this immaturity is the permanence of spontaneous pacemaker activity which is associated with the persistently high expression of the *I*_f_ current and the very low levels of *I*_K1_ [23]. Interestingly, however, these two features are the prototypical functional markers of sinoatrial cells. Thus, hiPSC-derived cardiomyocytes may represent a favourable model for studying human pacemaker activity of sinoatrial-like cells. For this reason, this paper characterized the *I*_f_ current restricting the electrophysiological recordings to only pCMs, that is those cells showing a regular spontaneous beating activity.

Under our experimental conditions, we observed that the *I*_f_ current density remained constant over time in culture. Our values are comparable with those previously reported in the literature both for hESC-CMs and iPSC-CMs [10, 30]; however, they are slightly smaller than those observed in diseased human SAN cells [39]. Of note, despite we did not employed any specific selection to enrich the culture in SAN-like myocytes, the mean *I*_f_ densities reported here for pCMs (− 4.3, − 5.8, − 5.2 at −125 mV, at days 15, 30, and 60 respectively) are compatible with the *I*_f_ density reported by Protze et al. in selected sinoatrial-like cells (~ 4.5 pA/pF at −120 mV) and much higher than that reported for ventricular-like cells (~ 1.5 pA/pF at −120 mV) [34]. This evidence suggests that the choice of spontaneously beating cells is a convenient and reliable method to select *bona fide* sinoatrial-like cells, when culture purity is not an issue.

Between day 15 and day 30, we observed a slightly but significant leftward shift of the *V*_1/2_ of the activation curves. A similar shift has been previously demonstrated in hESC due to a specific HCN4-Caveolin-3 interaction [10], shift that can be reverted by caveolae disruption with Methyl-β-cyclodextrin or by disruption of the caveolin-binding motif of HCN4 [6]. The mean *V*_1/2_ value reported in our study is within the physiological range for *I*_f_ to contribute to the DD, and close to the values reported for hESC-derived cardiomyocytes at similar maturation stages [10], and in mature rabbit SAN [3]. However, it differs by 20 mV from that reported for human SAN cells (around − 97 mV). This difference, and in particular a negative *V*_1/2_ may be due to methodological reasons (such as failure to reach current steady-state activation at depolarized voltages or from the presence of different extent of current run-down [20], and/or from the remodelling of the pathological human SAN cells analysed by Verkerk et al. [39].

The maturation of the properties of the funny current with time in pCMs is also indicated by changes in the *τ* of activation. pCMs at day 15 have significantly faster *τ* than at days 30 and 60. At these later stages, *τ* values are comparable with those found in the literature for rabbit SAN cells [3] and for human SAN cells [39]. Slower activation kinetics and more negative activation voltages (for the same current–density) would lead to a lower contribution of *I*_f_ during the slow diastolic depolarization and consequently a decreased rate. Accordingly, we found that spontaneous rate of pCMs significantly decreased from 1.27 ± 0.31 Hz at day 15 (*n* = 20) to 0.88 ± 0.47 Hz at day 30 (*n* = 26; *P* = 0.0025, data not shown).

Fully activated *I*-*V* relation of *I*_f_ shows a slight outward rectification and the expected reversal potential around −20 mV, compatible with the mixed Na^+^/K^+^ selectivity typical of both rabbit *I*_f_ [20] and heterologously expressed human HCN channels [29]. This is further confirmed by the dependence of *I*_f_ on the external Na^+^ and K^+^ concentrations. In agreement with data of DiFrancesco et al. in calf purkinje fibres [14] and in rabbit SAN cells[20], increasing [K^+^]_o_ induced a small positive shift of *E*_rev_ and a fourfold increase in conductance, while lowering [Na^+^]_o_ concentration resulted only in a more negative *E*_rev_. Furthermore, 2 mM Cs in the external solution reduced the inward component of *I*_f_ without affecting the conductance at potentials more positive than the *E*_rev_ [20].

In the human heart, HCN channels are widely distributed and their isoform expression ratio changes according with the type, function, and maturation of the cardiomyocytes. The conduction system, and in particular the sinus node of many mammalian species, expresses mostly HCN4 and HCN1, while the working myocardium expresses predominantly the HCN2 isoform [7]. In the mouse, HCN4 is the first isoform expressed during cardiogenesis when the primary myocardium forms, and later in development, its expression remains restricted to the sinoatrial node and the conduction system [2]. Here we show that pCMs express HCN4 and HCN1 mRNA as the predominant isoforms. These data are also in agreement with data obtained from SAN-like cells obtained after selection/enrichment procedure [34, 37] and from human SAN cells [13, 28].

It is now well established that the properties of the native *I*_f_ do not depend exclusively on the specific HCN subunits but also on their interaction with several accessory subunits, which influence channel trafficking, subcellular localization, and fine tune conductance and kinetics [35]. The accessory subunits MiRP1 SAP97, KCR1, and caveolin3 are all expressed in pCMs.

Finally, we provided here, for the first time, the whole dose–response curves of human *I*_f_ to autonomic stimulation. Increasing doses of isoproterenol progressively shifted the activation curve to more positive voltages with a maximum shift recorded of around 6 mV at saturating doses, a shift similar to that previously reported for rabbit SAN cells [4]. Hill fitting revealed an EC_50_ value close to that previously reported in rabbit SAN [40]. The muscarinic agonist carbachol progressively shifted the activation curve to more negative voltages with an EC_50_ of 11.6 nM, equal to that obtained in rabbit SAN [21, 40]. However, maximal shift with CCh was only of ~ 3 mV compared to the more than 6 mV reported in rabbit SAN [42]. We believe that the reason for the small effect of CCh in our pCMs is the low level of cAMP; indeed, pre-stimulation of the adenylate cyclase with 100 nM isoproterenol increased the response to 100 nM CCh to 6.4 mV compared to the 2.9 mV without pre-stimulation. These data clearly rule out the lack of expression of either muscarinic receptors or associated G proteins and that instead pCMs functionally express important proteins necessary for autonomic modulation of the *I*_f_ current and spontaneous rate. The low level of cAMP may for example derive from the high activity of phosphodiesterases (PDEs). The role of PDEs and in particular PDE3 and PDE4 in hiPS-CM has been recently shown in various works in which their inhibition resulted in the increase of basal cAMP level [22, 25, 26]. We may speculate that the low level of cAMP and high PDE activity may also explain the significantly slower time constant of the isoproterenol-mediated rate acceleration (7.6 ± 1.0 s) than the time constant of the rate slowing due to muscarinic receptors activation (3.4 ± 0.8 s).

Finally, we evaluated how these rate-modulators affect the beating activity of pCMs clusters; as expected and in line with the low cAMP levels, 1 µM Iso doubled the rate, while 100 nM ACh decreased it only by 13%; the same concentration of ACh applied to rabbit SAN cells led to a 68% decrease in the action potential rate [21]. Interestingly, a linear regression analysis of basal rate vs agonist-induced rate change revealed that, while the isoproterenol-induced increase in rate results mildly but significantly correlated to basal rate (Pearson’s *r* value =  − 0.56, *P* < 0.05), the Ach-induced decrease in rate is not correlated (Perason’s *r* value = 0.35, *P* = 0.43). All these data suggest that in our model, the muscarinic pathway is present and functional but because of the low basal cAMP levels, the response is blunted.

In conclusion, this work provides the first complete description of the properties of *I*_f_ in human-induced pluripotent stem cell (iPSC)–derived pacemaker cardiomyocytes. The set of kinetics and modulatory parameters provided here may represent useful comparative elements for future studies of cardiac diseases in which alterations of the *I*_f_ current or of HCN channels may play an important role in the onset of the pathology, as recently demonstrated [9].

## Data Availability

All data supporting the findings of this study are available within the article and from the corresponding author on reasonable request.
